# Yeast Stn1 promotes MCM to circumvent Rad53 control of the S phase checkpoint

**DOI:** 10.1007/s00294-022-01228-0

**Published:** 2022-02-12

**Authors:** Hovik Gasparayan, Chris Caridi, Jeff Julius, Wenyi Feng, Jeff Bachant, Constance I. Nugent

**Affiliations:** 1 Department of Molecular Cell Systems Biology, University of California, Riverside, Riverside, CA 92521 USA; 2 Department of Biological Sciences, University of Southern California, Los Angeles, CA 90089 USA; 3 Department of Biochemistry and Molecular Biology, State University of New York Upstate Medical University, Syracuse, NY 13210 USA

**Keywords:** Stn1, Rad53, MCM, S phase checkpoint, DNA replication origin, DNA replication stress

## Abstract

Treating yeast cells with the replication inhibitor hydroxyurea activates the S phase checkpoint kinase Rad53, eliciting responses that block DNA replication origin firing, stabilize replication forks, and prevent premature extension of the mitotic spindle. We previously found overproduction of Stn1, a subunit of the telomere-binding Cdc13–Stn1–Ten1 complex, circumvents Rad53 checkpoint functions in hydroxyurea, inducing late origin firing and premature spindle extension even though Rad53 is activated normally. Here, we show Stn1 overproduction acts through remarkably similar pathways compared to loss of *RAD53*, converging on the MCM complex that initiates origin firing and forms the catalytic core of the replicative DNA helicase. First, mutations affecting Mcm2 and Mcm5 block the ability of Stn1 overproduction to disrupt the S phase checkpoint. Second, loss of function *stn1* mutations compensate *rad53* S phase checkpoint defects. Third Stn1 overproduction suppresses a mutation in Mcm7. Fourth, *stn1* mutants accumulate single-stranded DNA at non-telomeric genome locations, imposing a requirement for post-replication DNA repair. We discuss these interactions in terms of a model in which Stn1 acts as an accessory replication factor that facilitates MCM activation at *ORI*s and potentially also maintains MCM activity at replication forks advancing through challenging templates.

**Supplementary Information:**

The online version contains supplementary material available at 10.1007/s00294-022-01228-0.

## Introduction

Cells must tolerate various forms of DNA replication stress, ranging from extrinsic mutagens to endogenous physiological perturbations. A particularly severe form of replication stress arises when cellular dNTPs are reduced; this stress can be experimentally induced using the ribonucleotide reductase (RNR) inhibitor hydroxyurea (HU). Reducing dNTPs slows advance of replication forks, greatly increasing the likelihood of replication fork collapse (Poli et al. 2012). The S phase checkpoint is a stress response pathway that is activated to safeguard against such catastrophes. In budding yeast, the S phase checkpoint consists of a core signaling axis of three protein kinases: Mec1, Rad53 and Dun1 (Giannattasio and Branzei 2017; Pardo et al. 2017). Mec1 is recruited to single-stranded DNA (ssDNA) that accumulates at stressed replication forks, after which Mec1 phosphorylates and activates Rad53. Rad53 then phosphorylates Dun1 to complete the signaling cascade. Dun1 plays a multi-faceted role in upregulating RNR, leading to expansion of dNTP pools (Zhou and Elledge 1993; Huang et al. 1998; Zhao and Rothstein 2002; Lee et al. 2008; Wu and Huang 2008). Rad53 controls other aspects of the checkpoint, including delaying activation of DNA replication origins (*ORI*) that normally fire later in the S phase program (referred to here as Rad53-checked *ORI*s; Santocanale and Diffley 1998; Shirahige et al. 1998; Feng et al. 2006), stabilizing replication forks (Lopes et al. 2001; Sogo et al. 2002; Cotta-Ramusino et al. 2005; Bermejo et al. 2011; Rossi et al. 2015; Colosio et al. 2016; Gan et al. 2017; Chappidi et al. 2019; Devbhandari and Remus 2020; Cabello-Lobato et al. 2021), and preventing premature extension of the bipolar mitotic spindle that assembles in HU-arrested yeast cells (Krishnan et al. 2004; Bachant et al. 2005; Julius et al. 2019). These responses synergize to allow DNA synthesis to proceed at a slow but steady rate in HU (Alvino et al. 2007; Poli et al. 2012; Zhong et al. 2013), to circumvent dNTP depletion (Morafraile et al. 2015), and to retain the capacity for accurate chromosome segregation once the extended S phase has been completed (Feng et al. 2009).

In previous work, we identified Stn1 as an additional protein connected to the budding yeast S phase checkpoint (Gasparyan et al. 2009). Stn1 was initially identified as a component of the conserved Cdc13–Stn1–Ten1 (CST) complex (Grandin et al. 1997; Rice and Skordalakes 2016). In yeast, CST binds telomere DNA repeats and protects chromosome ends from exonuclease digestion during S phase (Garvik et al. 1995; Nugent et al. 1996; Lin and Zakian 1996; Maringele and Lydall 2002; Jia et al. 2004; Bertuch and Lundblad 2004; Zubko and Lydall 2006; Vodenicharov and Wellinger 2006; Xu et al. 2009; Dewar and Lydall 2012; Langston et al. 2020). Yeast Stn1 also binds the Pol12 subunit of the DNA polymerase α/DNA primase complex (Polα; Grossi et al. 2004; Petreaca et al. 2006). The Stn1–Polα interaction plays a conserved role in chromosome end replication by stimulating Polα priming and fill-in synthesis of telomerase-generated ssDNA overhangs (Qi and Zakian 2000; Grossi et al. 2004; Petreaca et al. 2007; Puglisi et al. 2008; Chen and Lingner 2013). Importantly, however, other observations implicate CST in genome-wide aspects of DNA replication, particularly under conditions of DNA replication stress (Stewart et al. 2018). In a previous publication, we showed *STN1* overproduction (*STN1* OP) causes yeast cells to become extremely sensitive to HU and other replication stressors (Gasparyan et al. 2009). Remarkably, *STN1* OP also phenocopies *rad53* S phase checkpoint defects in HU, including activation of later-firing, Rad53-checked *ORI*s and premature spindle extension. Rad53 is activated normally in *STN1* OP cells, indicating upstream events in S phase checkpoint signaling are not perturbed by excess Stn1. While this suggests *STN1* OP acts downstream or in parallel to Rad53 to antagonize checkpoint effector responses, the underlying mechanisms remain to be defined.

Rad53 checks the firing of late *ORI*s through phosphorylation and inhibition of two proteins, Dbf4 and Sld3, required for activation and assembly of the Cdc45–GINS–MCM (CMG) replicative helicase (Lopez-Mosqueda et al. 2010; Zegerman and Diffley 2010; Duch et al. 2011). Dbf4 is a cyclin-like activator for Cdc7, the yeast Dbf4-dependent protein kinase (DDK; Jackson et al. 1993; Bousset and Diffley 1998). The DDK plays an essential role in *ORI* firing by phosphorylating paired Mcm2-7 hexamers (MCM) at licensed *ORI*s (Labib 2010). One consequence of MCM phosphorylation is to recruit Sld3 (Fang et al. 2016; Deegan et al. 2016). Sld3, in parallel, is phosphorylated by S phase forms of Cdk1 (S-CDK), leading to phospho-adapted interactions that recruit Cdc45 and GINS (Tanaka et al. 2007; Zegerman and Diffley 2007; Muramatsu et al. 2010). MCM activation corresponds with conformational changes that melt *ORI* DNA, with each strand entering the interior core of one MCM hexamer in the necessary configuration for bidirectional DNA unwinding (Georgescu et al. 2017; Douglas et al. 2018; Meagher et al. 2019). The DDK and S-CDK, thus, control parallel pathways activating MCM. Rad53 antagonizes both pathways in response to replication stress, imposing a robust check on further *ORI* firing.

An additional Rad53 checkpoint function circumvented by *STN1* OP is to prevent defective extension of the mitotic spindle during an extended S phase. Restraint of spindle extension has generally been considered to be controlled through a separate Rad53 cell cycle arrest pathway, unrelated to regulation of *ORI* firing and replication fork stabilization. In a recent study, however, we presented evidence that spindle extension in HU-treated *rad53* mutants is actually a consequence of a primary defect in DNA replication control (Julius et al. 2019). First, double mutant combinations predicted (*rad53 mcm2-1*, *rad53 mcm5-1*) or demonstrated (*rad53 dbf4-zn*) to reduce *ORI* firing in HU, or to suppress exonuclease processing of reversed replication forks (*rad53 exo1-∆*), suppressed the *rad53* spindle extension defect. Second, the *dbf4-zn* allele was preferentially defective for initiating *ORI* firing adjacent to centromeres (*CEN*s). Third, unregulated Exo1 activity in *rad53* mutants generated *CEN* ssDNA and perturbed kinetochore assembly. Based on these findings, we proposed that the critical role for Rad53 in restraining spindle extension in HU is to stabilize replication forks in proximity to *CEN*s. In the absence of this protective function, exonucleolytic degradation of *CEN* DNA disrupts kinetochore integrity and S phase spindle force balancing mechanisms. Here we have utilized this revised conception of the spindle extension defect in HU as a convenient genetic readout to assess pathways through which *STN1* OP antagonizes the S phase checkpoint. Our results indicate Stn1 is likely to act in concert with both the DDK and the MCM complex to efficiently activate *ORI* firing-a function revealed most prominently when *ORI*s fire in an unscheduled manner in the absence of the S phase checkpoint.

## Materials and methods

### Yeast culture

Relevant *S. cerevisiae* strains and plasmids are listed in figure legends. Cells were cultured in standard formulations of yeast extract/peptone/dextrose (YPD) and synthetic complete minimal (SC) media, with 2% glucose or 2% galactose as a carbon source. Cultures for microscopy were supplemented with 50 μg/mL adenine to quench autofluorescence associated with the *ade2* mutation in our strain backgrounds. For G_1_ synchronization/release, cells were treated with 10 μg/mL α-factor (Bio-Synthesis Corp.), typically for 1.5 h, washed in water, and released into desired culture media. Yeast transformation, strain construction, and other genetic manipulations were performed according to standard techniques (Guthrie and Fink 1991). HU was purchased from either Sigma-Aldrich or Fisher Scientific.  Yeast two-hybrid analysis was performed using strain PJ69-4a (James et al. 1996).

### Spindle length analysis

Spindle length distributions in fixed cell samples was performed as previously described (Bachant 2005). Cells harboring *SPC42-GFP* were released from G_1_ arrest into fresh media containing 200 mM HU. After 2.5 h, culture aliquots were briefly (1–5 min) fixed either using 1% formaldehyde diluted in phosphate buffered saline (PBS). Samples were washed into PBS and stored at 4 °C. DNA staining was performed using 4′6-diamidino-2-phenylindole (DAPI; Vecta-Shield, Vector Laboratories). Cells were visualized on either Nikon E-800 or Nikon Eclipse 80i microscopes equipped with florescence optics and 100 × (Plan Apo, 1.40 NA) objectives. The distance between Spc42-GFP spindle pole foci and bud circumference measurements were performed using the MetaMorph (Molecular Devices) suite of software tools.

### Western immunoblotting

To detect Stn1-HA in protein extracts, 25 mL cell cultures were grown to logarithmic phase (~ OD_600_ 0.8–1.0). Cells were harvested by centrifugation and lysed by bead beating (three 1 min bursts on a BioSpec BeadBeater 8) in 300 μL of 20% trichloroacetic acid (TCA) containing protease inhibitors (1 μg/mL leupeptin, 2 μg/mL aprotinin, 15 μg/mL benzamindine, 100 μg/mL PMSF, 10 μg/mL pepstatin). Lysates were centrifuged for 10 min at 3000 rpm at 4 °C to pellet proteins, and the TCA supernatant was removed. Protein pellets were resuspended in 100 μL of 1 M Tris base and 100 μL of Buffer A (25 mM HEPES, pH 7.5, 5 mM MgCl_2_, 50 mM KCl, 10% glycerol, 0.5% Triton X-100) supplemented with the protease inhibitor cocktail described above. 100 μL of 20% SDS and 60 μL of Laemmli sample buffer (50 mM Tris pH 6.8, 2% SDS, 10% glycerol, 0.1 M DTT, 0.01% bromophenol blue) were added to each sample, and the protein preparations were boiled at 95 °C for 5 min. 100 μL of each lysate was fractionated on 10% polyacrylamide gels and transferred to nitrocellulose membranes. The primary antibody (mouse anti-HA, 12CA5 from Roche) was used at a 1:1000 dilution in Tris-buffered saline (TBS) containing 3% non-fat dry milk, while the secondary antibody (HRP-conjugated goat anti-mouse from Chemicon) was used at a 1:25,000 dilution in TBS containing 3% non-fat dry milk.

### Chromosome spreads

To detect Stn1-HA on chromatin, 5 mL triplicate cell cultures were grown to logarithmic phase in appropriate selective media. Cells were collected by brief centrifugation and pellets were resuspended in 1 mL ZK buffer (25 mM Tris pH 7.5, 0.8 M KCl) supplemented with 40 μL of 1 M DTT, incubated for 2 min at room temperature. Samples were spheroplasted by addition of 5 μL of zymolyase solution (20 mg/mL zymolyase 100 T, 2% glucose, 50 mM Tris pH 7.5) and 2 μL of BME, and incubated for 15 min at 30 °C. The spheroplasted cells were washed with ice cold MES solution (1 M Sorbitol, 0.1 M MES pH 6.5, 1 mM EDTA, 0.5 mM MgCl_2_), then resuspended in 300 μL of MES solution. 20 μL of the cells were spotted onto a pre-cleaned glass slide, followed by addition of 40 μL of PFA solution (3% paraformaldehyde, 3.4% sucrose) and 80 μL of 1% lipsol. After 2 min of lysis, an additional 80 μL of PFA solution was added, and lysates were spread across the glass slides with a clean glass Pasteur pipette. Slides were dried at room temperature overnight. Prior to immunostaining, slides were washed with 0.2% Photoflo (Kodak) for 30 s and PBS for 5 min, then blocked with 350 μL of TBS containing 10 mg/mL BSA for 15 min at 4 °C. Excess blocking solution was drained, and 80 μL of primary antibody (mouse anti-HA, 12CA5 from Roche) was added at a 1:200 dilution in TBS containing 10 mg/mL BSA. Cover slips were applied, and slides were incubated at 4 °C in a wet chamber overnight. Slides were washed twice with TBS, drained, and 80 μL of secondary antibody (FITC-conjugated goat anti-rat from Sigma) was added at a 1:500 dilution in TBS containing 10 mg/mL BSA. Cover slips were added, and slides were stored in the dark from this point on. After a 2 h incubation at 4 °C, slides were washed with TBS twice, and dried at room temperature for 4 h. DNA was stained with DAPI as described above, cover slips were applied, and samples were visualized by fluorescence microscopy.

### Cell viability assays

5 mL cell cultures were grown to logarithmic phase. Cell concentration was determined by hemocytometery, after which cells were diluted into fresh media containing 200 mM HU. An aliquot of the culture was immediately removed and diluted as calculated so that 100 μL of the dilution yielded ~ 500 colony forming units when plated on solid media, providing an initial time point. At desired times, additional culture aliquots were removed, diluted in a similar fashion and plated to determine colony forming units. Colonies were counted after incubation for 3–5 days, and the fraction of surviving cells relative to the initial timepoint was determined.

### In situ Klenow primer extension on chromosomal ssDNA and Southern blotting

To detect ssDNA regions of chromosomes, we modified a previously described in situ ssDNA labeling method (Feng et al. 2011). Logarithmic phase cultures were washed and resuspended in 50 mM EDTA, and the cell concentration of each sample was determined by hemocytometery. For each sample, ~ 10^9^ cells were placed in a final volume of 500 μL. Cell suspensions were warmed to 55 °C and mixed with 500 μL of low melt agarose (Invitrogen) that had been dissolved in a 1:100 dilution of 1× TBE. The agarose/cell mixture was pipetted into plug molds and allowed to solidify at room temperature for 15 min. Solidified plugs were treated with 5 mL of spheroplasting solution (1 M sorbitol, 20 mM EDTA, 10 mM Tris pH 7.5, 14 mM BME, 0.5 mg/mL Zymolyase 20 T) for 4 h at 37 °C. Plugs were washed with SDS solution (1% SDS, 100 mM EDTA, 10 mM Tris pH8) twice for 15 min each, and incubated in SDS solution at 37 °C overnight with gentle rocking. The following day, plugs were washed with NDS solution (1% sarkosyl, 10 mM Tris base, 0.5 M EDTA pH 9.5) 3 times for 30 min each, followed by 5 washes with TE for 30 min each, then stored in 4 °C. Multiple plugs were prepared for each sample.

To perform the Klenow reactions, two plugs per sample were each pre-equilibrated in 5 mL of TMB (50 mM Tris pH 6.8, 5 mM MgCl_2_, 10 mM BME) for 30 min at room temperature. One plug was mixed with 400 μL of TMB buffer, 10 μL of dNTPs (10 μM each dNTP), 10 μL of random hexamer primers at 10 μM (Thermo Scientific), 100 units of Exo^−^ Klenow polymerase (New England BioLabs), and 50 μL of 10 × Klenow buffer. The second plug was treated identically, but no Klenow was added. The samples were incubated at 37 °C for 2 h and then washed with TE. Plugs were then pre-equilibrated with 1 × β-agarase buffer for 30 min on ice, heated to 65 °C to melt agarose, and treated with 5 units of β-agarase (New England BioLabs) for 1 h at 42 °C. The salt concentration was adjusted to 0.5 M NaCl, 0.8 M LiCl, 0.3 M NaO-Ac, samples were cooled on ice for 15 min, and DNA was precipitated with isopropanol. The DNA was then washed with cold 70% ethanol, dried, and resuspended in 40 μL of TE.

Prior to electrophoresis, reaction products were denatured by addition of 10 μL of 1 M NaOH and 1 μL of 0.5 M EDTA, followed by boiling at 95 °C for 5 min. Samples were cooled on ice for 5 min then electrophoresed on large format 1% agarose gel overnight at 50 V. DNAs were transferred to a nylon membrane (Hybond-XL, Amersham) overnight and crosslinked with 120 mJ of UV (Stratagene). The membrane was blocked with Church’s buffer (1% BSA, 1 mM EDTA, 0.5 M phosphate buffer, 7% SDS) overnight at 55 °C with gentle rocking. 25 μL of P^32^ radiolabeled TG_1–3_ probe was added, and the blot was incubated overnight at 55 °C with gentle rocking. The following day, the blot was washed three times with 1 L of washing solution (4 × SSC, 0.1% SDS), and exposed to X-ray film for 5 days at −80 °C. After development, the membrane was stripped by boiling in 0.1% SDS three times for 15 min each, then blocked with Church’s buffer as before. 25 μL of P^32^ radiolabeled rDNA probe was added, and the blot was processed for autoradiography as before.

## Results

### STN1 overproduction displays genetic interactions with DUN1, RNR2 and MCM

**Fig. 1:**
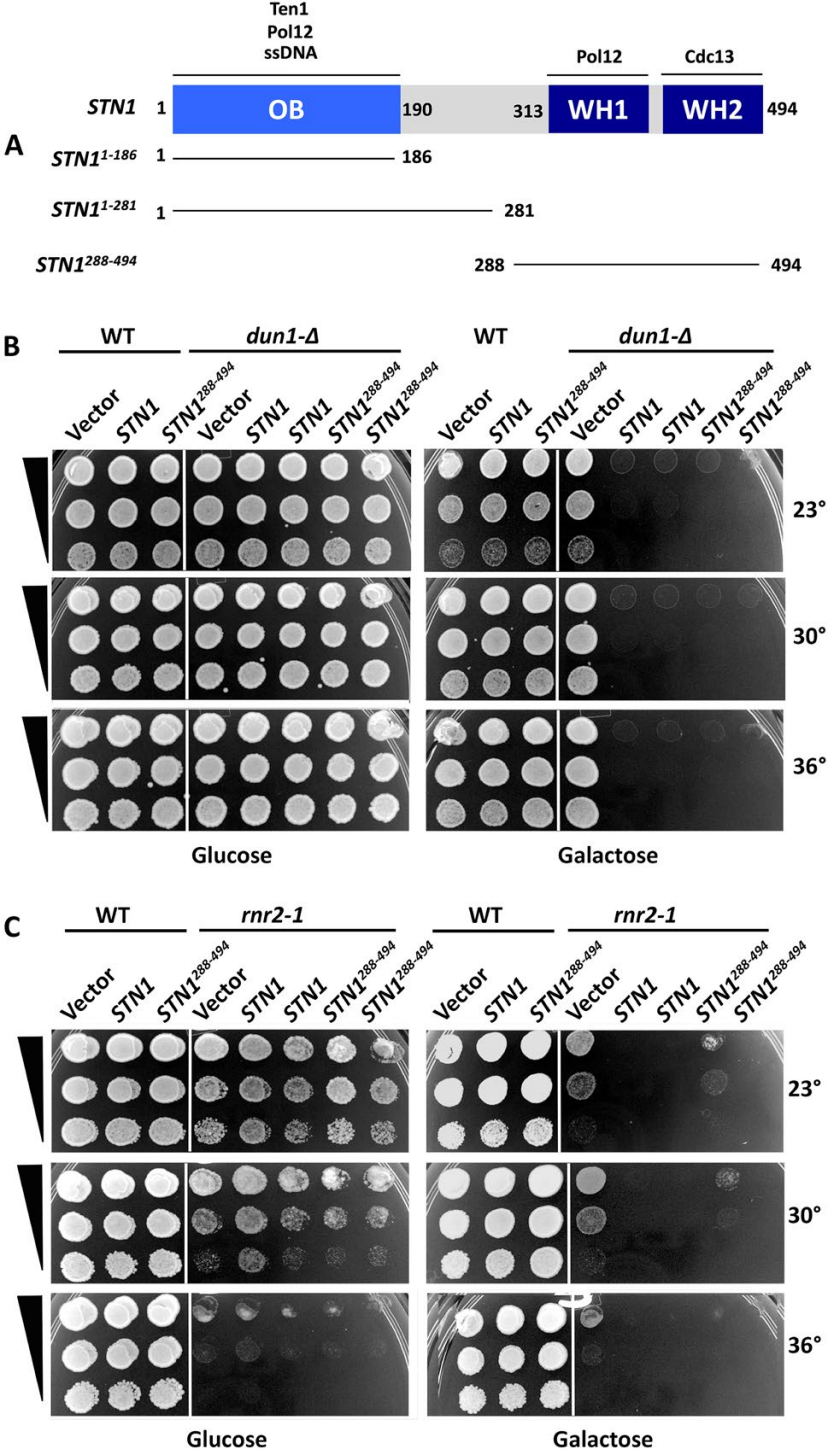
**STN1 OP is toxic to dun1-∆ and rnr2-1 mutants**. **A** The Stn1 protein includes an essential N-terminal OB fold domain and two winged helix (WH) domains at the C-terminus. Known protein of nucleic acid interactions mapping to these domains are indicated. *stn1*^1−186^ is a truncation allele that only expresses the first 186 codons. *stn1*^1−186^ and *stn1*^288−494^ refer to OP constructs that encode the indicated fragments of the Stn1 protein. In all three cases, lines in diagram indicate *STN1* codons that are expressed. **B** WT (Y300) or *dun1-∆* (Y286) cells were transformed with Vector, p*GAL-STN1* (*STN1* on figure) and p*GAL-stn1*^*288−494*^ (*stn1*^*288−494*^ on figure). Transformants were grown to saturation in selective media. Tenfold serial dilutions (black triangles) were stamped onto selective glucose or galactose solid media containing the indicated concentrations of HU. Duplicate sets of plates were cultured at 23°, 30° and 36 °C. **C** *rnr2-1* (Y221) cells were transformed and analyzed as in (**B**)

To identify genetic pathways affected by *STN1* OP, we chose an approach based on our previous finding that *STN1* OP checkpoint defects could be suppressed in a *pol12–40* mutant (Gasparyan et al. 2009). The Pol12–40 mutant protein is partially defective for binding Stn1 (Petreaca et al. 2006; see schematic of Stn1 domains and interactions, Fig. 1A), and retention of excess Stn1 on chromatin spreads is greatly reduced in *pol12–40* mutants (Gasparyan et al. 2009), implying *STN1* OP acts through *POL12*. Extending this logic, we over-expressed *STN1* under control of the galactose-inducible *GAL* promoter in a collection of mutants defective for DNA replication control or tolerating DNA replication stress. Transformants were evaluated on galactose media for enhancement/suppression of mutant growth defects or enhancement/suppression of *STN1* OP HU sensitivity. One interaction we identified was that *STN1* OP was synthetically lethal with a *dun1-∆* mutant, even in the absence of HU (Fig. 1B). Synthetic lethality with *dun1-∆* was also observed following OP of a Stn1 fragment (Stn1^288−494^; amino acids 288–494) comprising two C-terminal winged helix domains (Fig. 1A). *stn1*^*288−494*^ OP was assessed because this is the minimal *STN1* region required for OP checkpoint phenotypes (Gasparyan et al. 2009). We additionally examined the effect of *STN1* and *STN1*^*288−494*^ OP in a recessive loss of function *rnr2-1* temperature sensitive mutant (Zhou and Elledge 1992). OP of *STN1* and *STN1*^*288−494*^ strongly inhibited *rnr2-1* growth on galactose media, even at a permissive temperature of 23 °C (Fig. 1C). These observations suggest *STN1* OP imposes an essential requirement for Dun1-mediated upregulation of RNR.

**Fig. 2:**
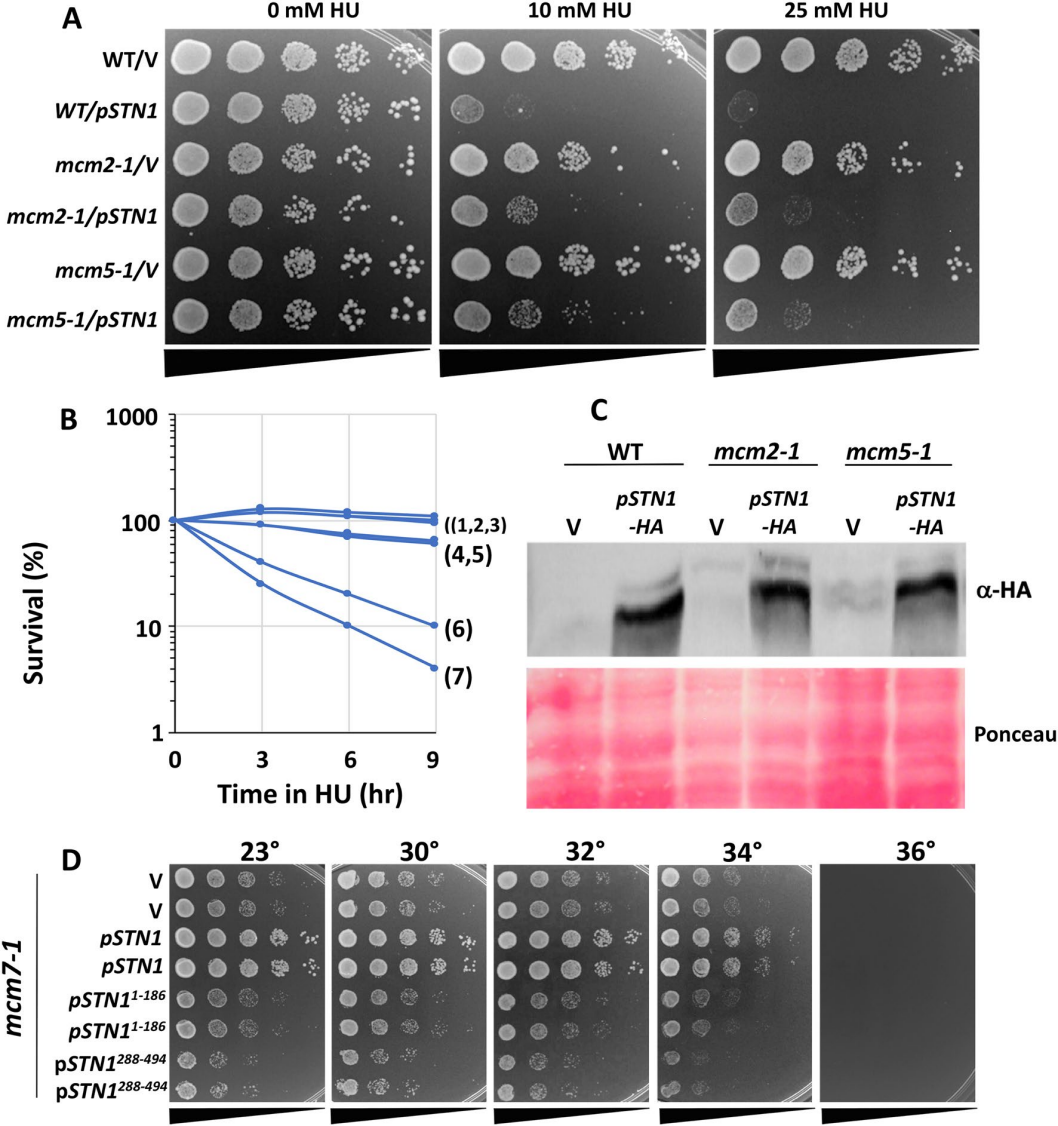
**Genetic interactions between STN1 and MCM2–7**. **A** WT/Vector (hc2110), WT/p*ADH-STN1* (p*STN1* or p*STN1-HA* on figure, hc2109), *mcm2-1*/Vector (hc2425), *mcm2-1*/p*ADH-STN1* (hc2426), *mcm5-1*/Vector (hc2427) and *mcm5-1*/p*ADH-STN1* (hc2428) strains were grown to saturation in selective media. Tenfold serial dilutions (black triangles) were stamped onto selective media containing the indicated concentrations of HU at 30°. The ADH promoter induces high levels of transcription and is constitutively active in glucose media. **B** Strains in (**A**), along with a *rad53-21* control (hc27), were grown to logarithmic phase in selective media and diluted into fresh media containing 200 mM HU (*T* = 0) and incubated at 30°. At indicated times aliquots were plated on media lacking HU to quantify recovery. Legend: 1, WT/V; 2, *mcm2-1*/V; 3, *mcm5-1*/V; 4, *mcm2-1*/p*ADH-STN1*; 5, *mcm5-1*/p*ADH-STN1*; 6, WT/p*ADH-STN1*; 7, *rad53-21*. **C** Strains in (**A**) were grown to logarithmic phase in selective media at 30°. Protein extracts were analyzed by immunoblotting with α HA to detect exogenous OP Stn1. **D** *mcm7-1* cells were transformed with Vector, p*ADH-STN1* (p*STN1* on figure), p*ADH-stn1*^*1−186*^ (p*stn1*^*1−186*^ on figure) or p*ADH-stn1*^*288−494*^ (p*stn1*^*288−494*^ on figure) plasmids. Tenfold serial dilutions of saturated cultures were stamped and incubated at indicated temperatures

We also identified genetic interactions between *STN1* OP and *mcm2-1*, *mcm5-1* and *mcm7-1*, recessive, temperature sensitive alleles that compromise MCM activity and DNA synthesis (Tye 1999). As MCM is a multimeric complex, it is notable that both *mcm* mutations and altered *MCM* expression produce a complex assortment of genetic interactions, including co-suppression and dosage enhancement (Yan et al. 1991). Additionally, *MCM7* has a second function as a cell cycle regulated transcriptional repressor, with the *mcm7-1* mutation increasing the expression of other *MCM* genes (Fitch et al. 2003). Against this backdrop, we observed that *STN1* OP in *mcm2-1* and *mcm5-1* partially alleviated the extreme HU sensitivity associated with *STN1* OP, allowing growth at up to 25 mM HU at a *mcm* semi-permissive temperature of 30 °C (Fig. 2A). Moreover, *mcm2-1* and *mcm5-1 STN1* OP transformants showed a marked improvement in their ability to recover from transient exposure to 200 mM HU at 30 °C (Fig. 2B). Immuno-blotting revealed similar amounts of OP Stn1 accumulated in WT, *mcm2-1* and *mcm5-1* transformants (Fig. 2C). The ability of *mcm2-1* and *mcm5-1* to allow *STN1* OP cells to recover from transient HU exposure is notable, as our prior work indicates that a dramatic loss of survival following acute HU exposure is typically a consequence of simultaneously deregulating *ORI* firing and destabilizing replication forks (Desany et al. 1998; Alcasabas et al. 2001; Julius et al. 2019).

As a further connection between *STN1* and *MCM*, we found *STN1* OP acted as a dosage suppressor of *mcm7-1* (Fig. 2D); a similar suppression of *mcm2-1* or *mcm5-1* was not observed (not shown). *mcm7-1* suppression did not occur following OP of either N- or C-terminal Stn1 regions, indicating the effect required full length Stn1 (Fig. 2D). In sum, these results indicate *STN1* OP exhibits complex interactions with MCM. On the one hand, reduced MCM function alleviates *STN1* OP HU toxicity. On the other, *STN1* OP partially restores viability to at least one MCM loss of function mutant strain.

### STN1 OP acts through MCM to induce S phase checkpoint defects

**Fig. 3:**
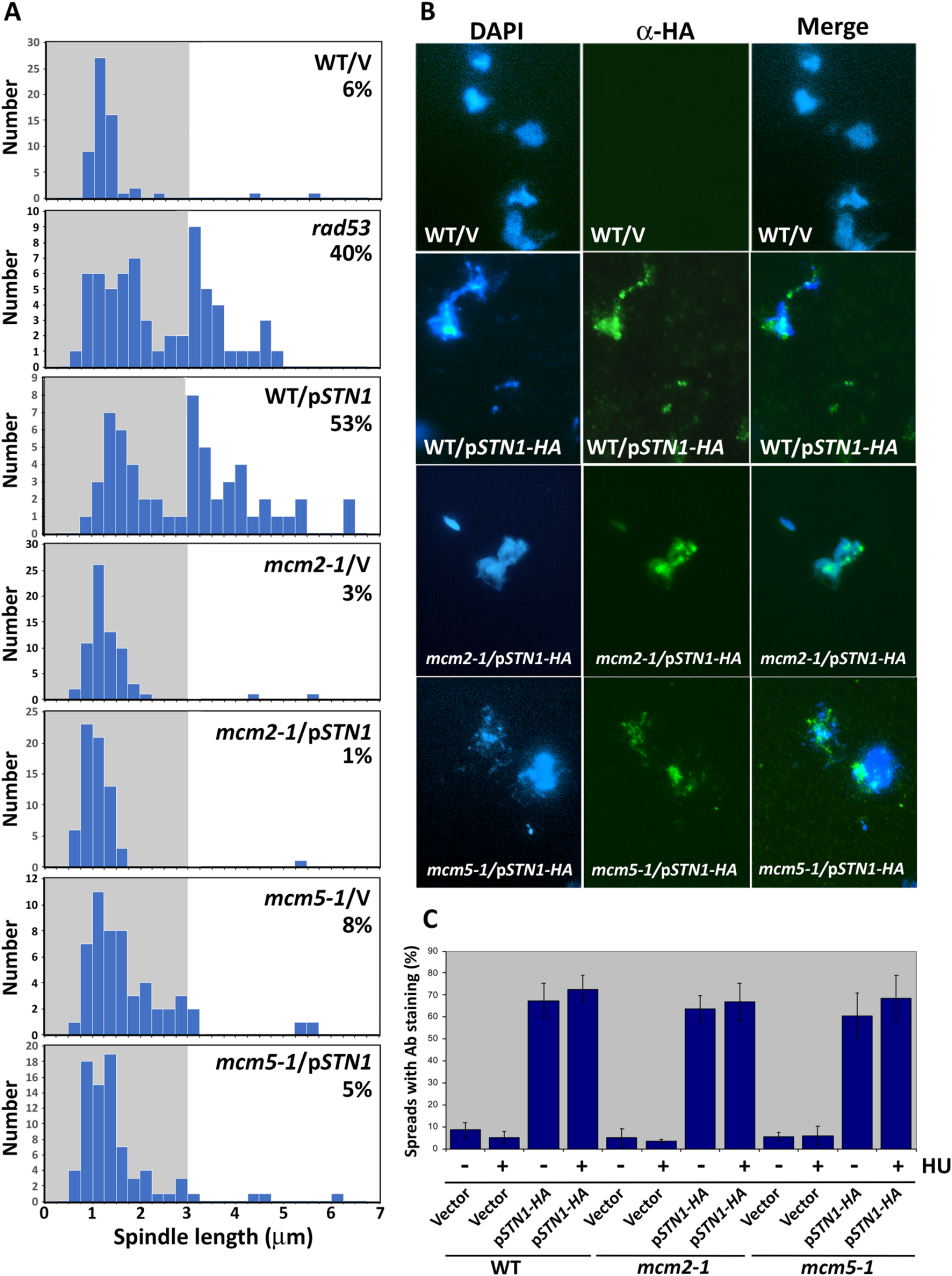
**mcm2-1 and mcm5-1 suppress STN1 OP-induced spindle extension in HU**. **A** WT/Vector (hc2110), WT/p*ADH-STN1* (p*STN1* or p*STN1-HA* on figure, hc2109), *mcm2-1*/Vector (hc2425), *mcm2-1*/p*ADH-STN1* (hc2426), *mcm5-1*/Vector (hc2427), *mcm5-1*/p*ADH-STN1* (hc2428) and *rad53-21* (hc27) strains harboring *SPC42-GFP* were arrested in G_1_ and released into 200 mM HU at 30 °C. At 2.5-h post-release, the distance between Spc42-GFP spindle pole foci was evaluated in ≥ 100 cells. Numbers on each histogram show percentage of spindles ≥ 3 μm. **B** Strains in (**A**) were cultured in selective media to logarithmic phase at 30 °C, lysed, and chromatin was spread on glass slides. Stn1 localization was monitored by α HA immunofluorescence and DNA counterstaining with DAPI. Micrographs show representative images. **C** Quantification of Stn1 OP chromatin binding. For each sample in (**B**), at least 100 DAPI-positive spreads were scored for α-HA Stn1 staining. Graph depicts average of three experiments ± one standard deviation

As described in the Introduction, we previously characterized *mcm2-1* and *mcm5-1* as mutations that suppressed the spindle extension phenotype of HU-treated *rad53* mutants (Julius et al. 2019). To see if a similar relationship was observed with *STN1* OP, WT, *mcm2-1* and *mcm5-1 STN1* OP strains were released from a G_1_ block into media containing 200 mM HU. Spindle length distributions were evaluated in fixed cells after 2.5 h using a GFP tagged spindle pole body protein (Spc42-GFP; representative micrographs of WT, *rad53* and *STN1* OP spindle morphologies in HU can be found in (Bachant et al. 2005; Gasparyan et al. 2009; Julius et al. 2019). As expected, WT cells transformed with a vector control displayed the short (1–2 μm) spindles characteristic of HU-arrested cells (% spindles ≥ 3 μm = 6), while 53% of WT/p*STN1* transformants exhibited a heterogenous range of spindle lengths ≥ 3 μm (Fig. 3A; 3 μm is our threshold for an extended spindle). In comparison*, mcm2-1*/p*STN1* and *mcm5-1*/p*STN1* transformants exhibited 1% and 5% spindle extension, respectively (Fig. 3A), a similar extent of suppression to that of HU-treated *mcm2-1 rad53* and *mcm5-1 rad53* strains (Julius et al. 2019). Unlike the situation with *pol12-40* suppression of *STN1* OP defects (Gasparyan et al. 2009), *mcm2-1* and *mcm5-1* did not reduce Stn1 binding to spread chromatin preparations (Fig. 3B).

**Fig. 4:**
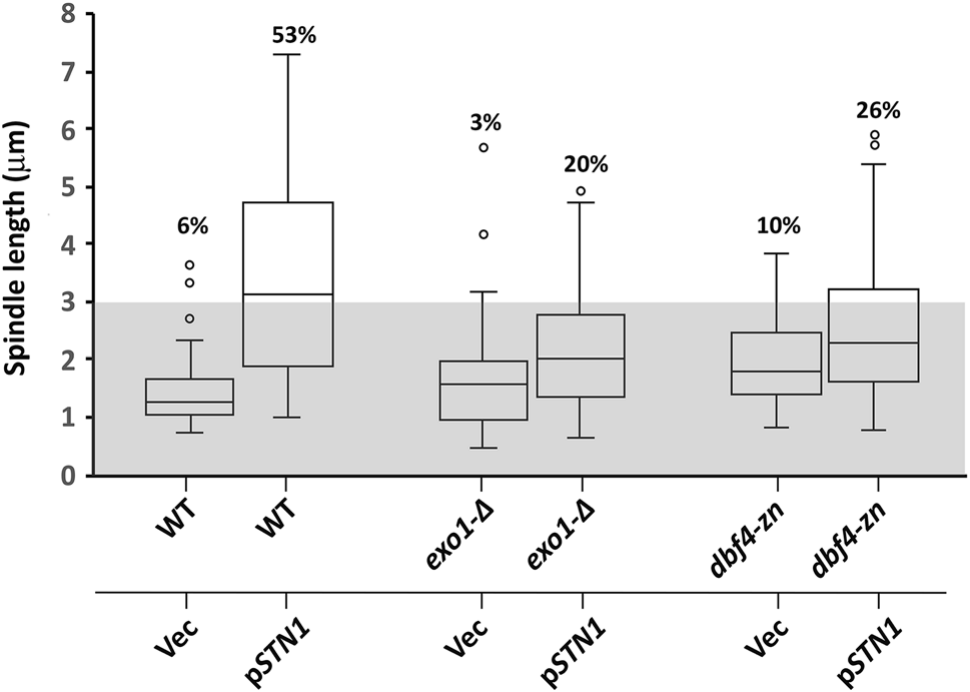
**Exo1-∆ and dbf4-zn suppression of STN1 OP-induced spindle extension in HU**. WT/Vector (hc2110), WT/p*ADH-STN1* (p*STN1* on figure, hc2109), *exo1-∆*/Vector, *exo1-∆*/p*ADH-STN1*, *dbf4-zn*/Vector (JJY063, JJY065), and *dbf4-zn*/p*ADH-STN1* (JJY064, JJY066) strains harboring *SPC42-GFP* were arrested in G_1_ and released into 200 mM HU at 30 °C. After 2.5 h post-release, the distance between Spc42-GFP spindle pole foci was evaluated in ≥ 100 cells. Box and whisker plots show spindle length distributions. Numbers above each plot show percentage of spindles ≥ 3 μm. The WT/Vector and WT/p*ADH-STN1* distribution is from the same experiment shown in Fig. 3A

To further compare the genetic requirements for spindle extension in HU-treated *rad53* and *STN1* OP cells, p*ADH*-*STN1* was transformed into *exo1-∆* and *dbf4-zn* strains. Previously, we found *rad53 exo1-∆* and *rad53 dbf4-zn* double mutants reduced the percentage of HU-treated cells with extended spindles by  ~ threefold and  ~sevenfold, respectively, compared to *rad53* controls (Julius et al. 2019). In response to *STN1* OP in HU, 20% of *exo1-∆*/p*STN1* cells exhibited spindles ≥ 3 μm, a 2.6-fold reduction compared to WT/p*STN1* (Fig. 4). Thus, loss of *EXO1* suppresses spindle extension in *rad53* and *STN1* OP cells to a fairly similar extent. With respect to the effect of *dbf4-zn*, 26% of HU-treated *dbf4-zn*/p*STN1* cells showed spindles ≥ 3 μm (Fig. 4). While this is a significant (*p* < 0.001, *t*-test) twofold reduction compared to WT/p*STN1*, the suppressive effect of *dbf4-zn* on *STN1* OP was not as extensive as what we had observed for *dbf4-zn rad53*. Overall, however, these results reveal a remarkable congruence in the genetic requirements for spindle extension in HU-treated *rad53* and *STN1* OP cells. Thus, loss of *RAD53* and gain of *STN1* appear to act through similar mechanisms to induce spindle extension.

### Loss of STN1 function suppresses rad53 S phase checkpoint defects

**Fig. 5:**
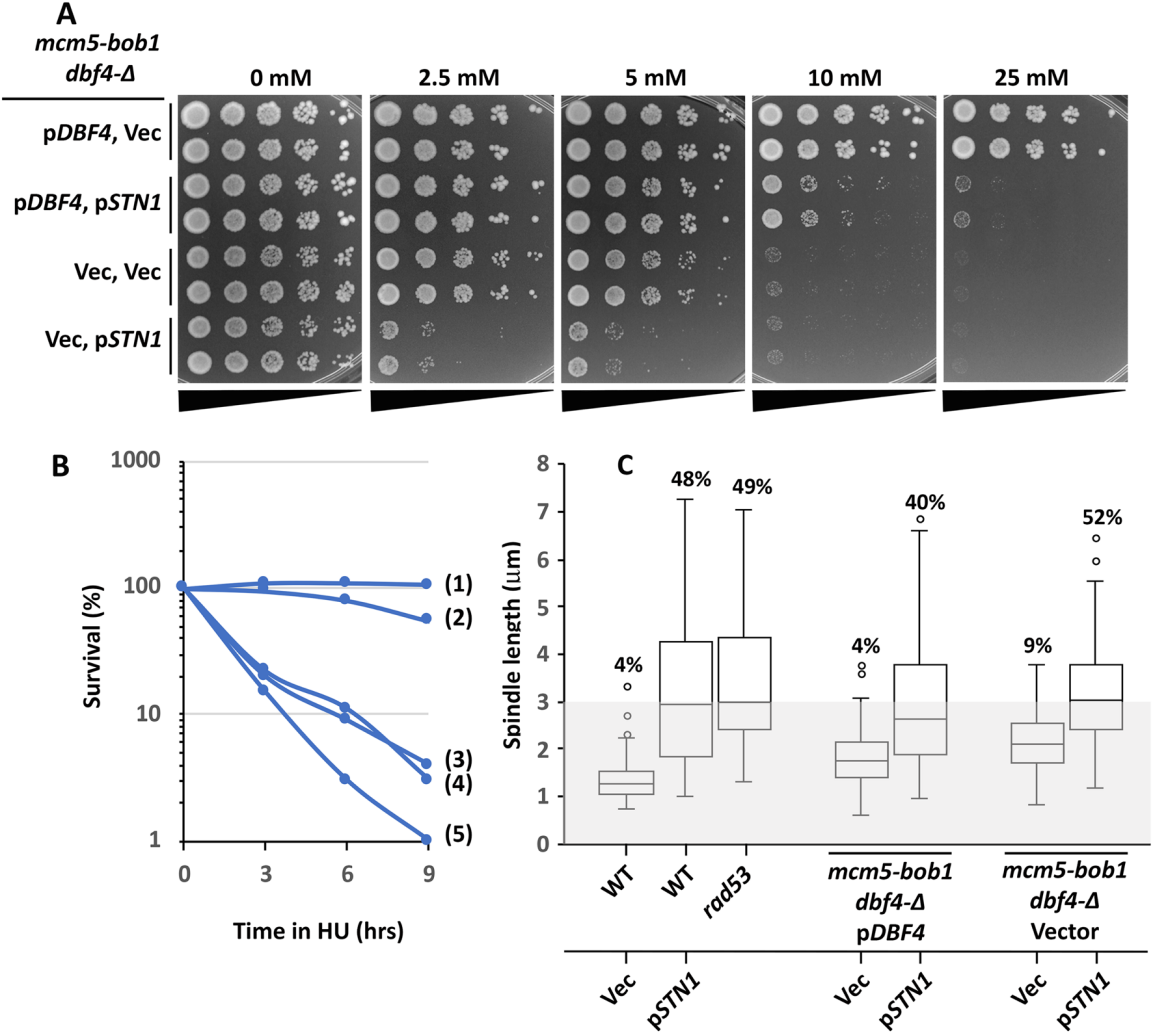
**DBF4 is not required for STN1 OP to antagonize the S phase checkpoint**. **A** *mcm5-bob1 dbf4-∆* was transformed with either a vector control (Vec) or a low copy plasmid expressing *DBF4* under control of the native promoter (p*DBF4*). The strains where then transformed with either a vector control (Vec) or p*ADH-STN1* (p*STN1* on figure) for *STN1* OP. *mcm5-bob1 dbf4-∆*/p*DBF4*, Vec (hc2405); *mcm5-bob1 dbf4-∆*/p*DBF4*, p*ADH-STN1* (hc2406); *mcm5-bob1 dbf4-∆*/Vec, Vec (hc2407); and *mcm5-bob1 dbf4-∆*/Vec, p*ADH-STN1* (hc2408) strains were cultured to saturation in selective media. Tenfold serial dilutions (black triangles) were stamped onto plates containing the indicted concentrations of HU and incubated at 30 °C. **B** Strains in (**A**), along with a *rad53-21* control (hc27) were grown to logarithmic phase and diluted into fresh media containing 200 mM HU (*T* = 0) at 30 °C. Culture aliquots were removed at indicated times and plated onto media lacking HU to quantify recovery. Legend: 1, *mcm5-bob1 dbf4-∆*/p*DBF4*, Vec; 2, *mcm5-bob1 dbf4-∆*/Vec, Vec; 3, *mcm5-bob1 dbf4-∆*/p*DBF4*, p*ADH-STN1*; 4, *mcm5-bob1 dbf4-∆*/Vec, p*ADH-STN1*; 5, *rad53-21*. **C** Strains in (**A**), along with WT/Vec (hc2110), WT/p*STN1* (hc2109) controls, all containing *SPC42-GFP*, were arrested in G_1_ and released into 200 mM HU media. The distance between Spc42-GFP spindle pole foci was evaluated in ≥ 100 cells at 2.5 h post-release. Spindle length distributions are represented as box and whisker plots. Numbers above each plot show percentage of spindles ≥ 3 μm

The results presented so far are consistent with the idea that *STN1* OP activates the MCM complex to induce firing of checked *ORI*s in HU. In this regard, it is interesting that OP of *DBF4* is similar to OP of *STN1* in being sufficient to circumvent Rad53 control of both checked *ORI* firing and spindle extension in HU (Mantiero et al. 2011; Tanaka et al. 2011; Julius et al. 2019). Furthermore, in searching for potential physical interactions between Stn1 and DNA replication factors we identified a two-hybrid interaction between Stn1 and Dbf4 (Supplemental Fig. 1). From this, one possibility is that excess Stn1 circumvents the Rad53 check on *ORI* firing in HU through DDK activation of MCM, prompting us to examine the consequences of eliminating *DBF4* in *STN1* OP cells. Such a genetic test is possible using a gain of function *mcm5* allele, *mcm5-bob1*, that bypasses the requirement for the DDK in *ORI* firing, allowing cells to proliferate in the absence of either Dbf4 or Cdc7 (Hardy et al. 1997; Hoang et al. 2007; Miller et al. 2014). Importantly, however, the Rad53 check on late *ORI* firing remains largely intact in *mcm5-bob1* mutants due to the necessity of also circumventing Rad53 inhibition of Sld3 (Zegerman and Diffley 2010), and ~ 90% of *mcm5-bob1* cells arrest with short spindles in HU (Fig. 5C). Thus, *STN1* OP in *dbf4-∆ mcm5-bob1* permits an epistatic test of whether *STN1* OP defects are directed exclusively through *DBF4*.

We observed *mcm5-bob1 dbf4-∆* cells displayed considerable sensitivity to HU, failing to grow at 10 mM HU (Fig. 5A). This sensitivity, however, was further exacerbated by *STN1* OP, with *mcm5-bob dbf4-∆*/p*STN1* cells exhibiting only weak growth on 2.5 mM HU. Additionally, whereas *mcm5-bob1 dbf4-∆* and *mcm5-bob1 dbf4-∆*/p*DBF4* cells largely recovered following transient 200 mM HU treatment, *mcm5-bob1 dbf4-∆*/p*DBF4,* p*STN1* and *mcm5-bob1 dbf4-∆*/p*STN1* cells failed to recover, exhibiting a defect that was comparable, although not quite as severe, as that displayed by *rad53* mutants (compare strains 3, 4 with strain 5, Fig. 5B). As described above, a dramatic loss of viability following acute exposure to HU is indicative of S phase checkpoint deregulation, associated with unscheduled *ORI* firing and replication fork catastrophes. Associated with HU sensitivity, 9% of *mcm5-bob1 dbf4-∆* cells displayed extended spindles in HU, a slight (but significant, *p* < 0.001, *t*-test) increase over *mcm5-bob1 dbf4-∆*/p*DBF4* controls (Fig. 5C). In comparison, 52% of HU-treated *mcm5-bob1 dbf4-∆*/p*STN1* cells, 40% of *mcm5-bob1 dbf4-∆/*p*DBF4*, p*STN1*, and 48% of *MCM5 DBF4*/p*STN1* cells displayed extended spindles (Fig. 5C). The spindle length distributions of *mcm5-bob1 dbf4-∆*/p*STN1*, *mcm5-bob1 dbf4-∆/*p*DBF4*, p*STN1*, and *MCM5 DBF4*/p*STN1* were all statistically comparable (*mcm5-bob1 dbf4-∆*/p*STN1* vs. *mcm5-bob1 dbf4-∆/*p*DBF4*, p*STN1*, *p* = 0.15; *mcm5-bob1 dbf4-∆*/p*STN1* vs. *MCM5 DBF4*/p*STN1* = 0.53; *mcm5-bob1 dbf4-∆/*p*DBF4*, p*STN1* vs. *MCM5 DBF4*/p*STN1* = 0.12). To summarize: (1) Stn1 interacts with Dbf4 in the two-hybrid assay; (2) *dbf4-zn* partially alleviates *STN1* OP spindle extension in HU; and (3) the absence of *DBF4* in *mcm5-bob1* does not suppress *STN1* OP in HU compared to *mcm2-1* and *mcm5-1*. Thus, the DDK cannot be the only target of *STN1* OP.

**Fig. 6:**
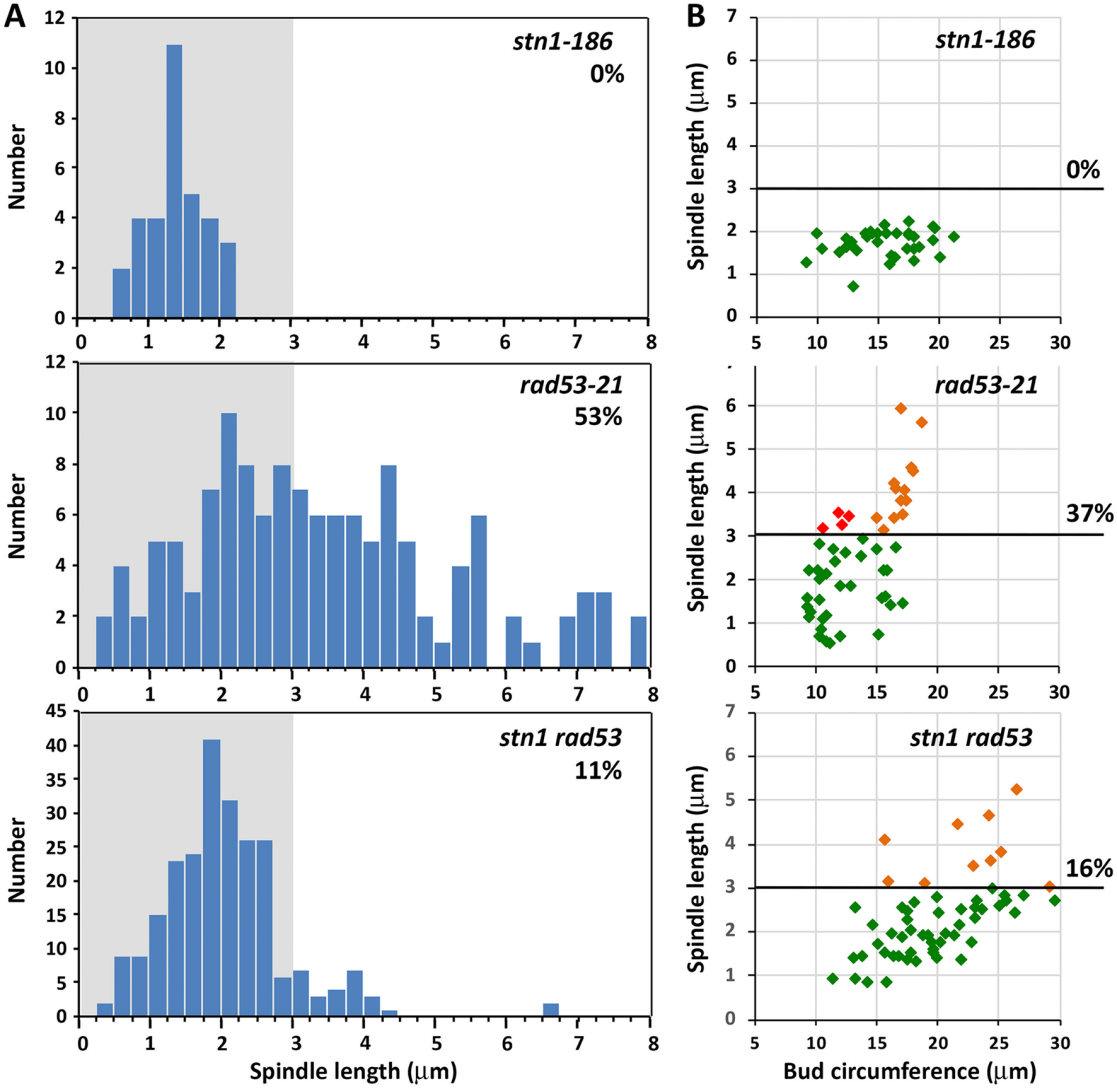
**stn1**^**1–186**^** is a suppressor of rad53 spindle extension in HU**. **A** *stn1*^*1−186*^, *rad53-21* (hc2804) and *rad53-21 stn1*^*1−186*^ (hc2806) strains harboring *SPC42-GFP* were released from a G_1_ arrest into 200 mM HU media. The distance between Spc42-GFP spindle pole foci was evaluated at 2.5-h post-release. Numbers on histograms indicate the percentage of cells with spindles ≥ 3 μm. **B** The same strains were processed as in (**A**), except in this experiment both bud circumference (as a metric for elapsed time in S phase) and spindle length were quantified. Color coding on graphs: cells with spindles ≤ 3 μm, green; cells with spindles ≥ 3 μm and bud circumferences ≤ 15 μm (small- to medium-budded cells), red; cells with spindles ≥ 3 μm and buds ≥ 15 μm (medium- to large-budded cells), orange. The percentage of total cells with extended spindles is shown on the right-hand side of each graph

If *STN1* acts through a partially separable pathway from the DDK to activate MCM, an additional genetic test is to ask whether loss of Stn1 acts similarly to *mcm2-1* and *mcm5-1* in suppressing *rad53* phenotypes. *stn1*^*1−186*^ is a loss of function truncation allele expressing the first 186 N-terminal codons of *STN1* (Petreaca et al. 2007) (Fig. 1A). *stn1*^*1−186*^ fails to suppress the temperature sensitivity of *mcm7-1*, suggesting it defective for this aspect of *STN1* function (Fig. 2D). Moreover, *stn1*^*1−186*^ mutants arrest in HU with short spindles, indicating they are proficient for the S phase checkpoint (Fig. 6A, B). We therefore constructed a *rad53-21 stn1*^*1−186*^ double mutant and evaluated spindle extension in HU. We observed *rad53-21 stn1*^*1−186*^ mutants exhibited 11% spindle extension compared to 53% for *rad53-21*, a significant reduction (*p* < 0.001, *t*-test, Fig. 6A). In a related experiment we correlated spindle length with bud circumference in HU-treated cells, using bud circumference as a metric for elapsed time in S phase. HU-treated *rad53* mutants typically initiate spindle extension shortly after S phase entry, when bud circumference is ~ 10–12 μm (Julius et al. 2019). This early period of spindle extension was completely rescued in *rad53-21 stn1*^*1−186*^, suggesting a restoration in the delay of spindle extension (Fig. 6B). *STN1,* therefore, appears to be a genetic effector of the spindle extension phenotype.

**Fig. 7:**
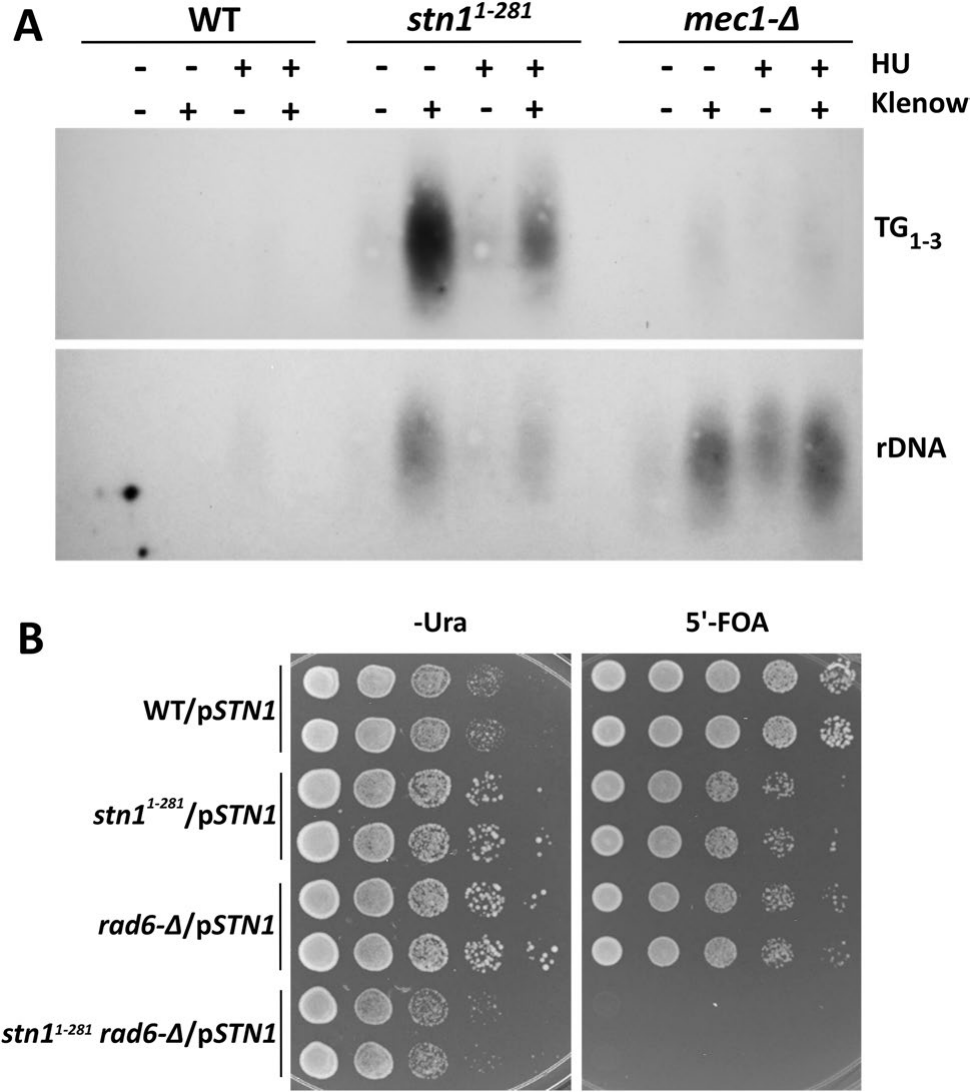
**Accumulation** **of** **ssDNA** **damage** **in** *stn1* **mutants**. **A** WT (hc160), *stn1*^*1−281*^ (hc671) and *mec1–21* (hc30) strains were grown to logarithmic phase at 30 °C or additionally treated with media containing 200 mM HU for 3 h. For each sample, a fixed number of cells were suspended in agarose plugs, spheroplasted, and either treated or not treated with hexameric primers and Klenow DNA polymerase. Extension products corresponding to chromosomal ssDNA were analyzed by Southern blotting with either telomeric (upper panel, TG_1–3_) or rDNA (lower panel) probes. **B** WT (hc160), *stn1*^*1−281*^ (hc671), *rad6-∆* (JBY285), *stn1*^*1−281*^*rad6-Δ* (hc2636) strains all harboring p*STN1-URA3* (pVL1046) were grown to saturation in selective media and tenfold serial dilutions were stamped onto either Ura^−^ or 5’-FOA containing media. Plates were incubated for 4 days at 30 °C

*stn1* mutants accumulate ssDNA at telomeres, arising from defects in telomere replication and chromosome end protection (Grandin et al. 1997). If Stn1 functions more globally in DNA replication, we hypothesized Stn1 might also prevent accumulation of ssDNA at interior chromosomal regions. To test this, we modified a previously described procedure for in situ labeling of chromosomal ssDNA (Feng et al. 2011). *stn1*^*1−281*^, WT and *mec1–21* cells were embedded in agarose, permeabilized, and DNAs complementary to ssDNA regions were synthesized using random oligonucleotide primers and Klenow DNA polymerase. After a denaturation step and nucleic acid recovery, short primer extension products were separated from the larger mass of chromosomal DNA by electrophoresis and analyzed by Southern blotting. As expected, *stn1*^*1−281*^, but not WT or *mec1* controls, displayed a ssDNA signal when the blots were probed with a telomeric DNA repeat sequence (TG_1–3_ panel, Fig. 7A). Re-probing the blot with a repetitive sequence within the rDNA locus revealed *stn1*^*1−281*^ also accumulated ssDNA at this internal chromosomal region (rDNA panel, Fig. 7A). ssDNA at the rDNA locus was also observed in *mec1–21*, which is known to accumulate replicative DNA damage (Feng et al. 2011). Treating *stn1*^*1−281*^ cells with HU showed that blocking DNA synthesis considerably reduced both telomeric and rDNA signals. In contrast, *mec1*−*21* cells, which fail to check *ORI* firing and experience replication catastrophes in HU, did not display such a reduction. HU-treated *mec1*−*21* samples also showed a fainter rDNA signal in the absence of Klenow (Fig. 7B). To explain this, chromosome fragmentation and nuclease assault in HU-treated *mec1* cells (Cha and Kleckner 2002; Feng et al. 2009, 2011) may generate rDNA fragments that are small enough to enter the gel and be visualized by our method. *stn1*^*1−281*^ was used for the experiment shown in Fig. 7A because this allele causes a more severe growth defect than *stn1*^*1−186*^. However, *stn1*^*1−186*^ was subsequently found to also accumulate ssDNA at the rDNA locus (not shown).

To determine if ssDNA accumulation in *stn1* mutants was physiologically relevant, we examined whether Rad6-dependent post-replication DNA repair was required for the viability of *stn1* cells. Initial crosses indicated it was not possible to isolate viable *stn1*^*1−281*^* rad6*-∆ double mutant segregants. We repeated this analysis using a *stn1*^*1−281*^ parental strain harboring *STN1* on a low copy *URA3* plasmid, allowing *stn1*^*1−281*^* rad6*-∆/p*STN1-URA3* segregants to be obtained. *stn1*^*1−281*^* rad6*-∆ double mutants harboring p*STN1-URA3* failed to grow on 5’-FOA containing media, which selects against cells unable to lose the *URA3* plasmid. The dependency of *stn1*^*1−281*^* rad6*-∆/p*STN1-URA3* strains on the covering *STN1* plasmid indicates *stn1* mutants require Rad6-mediated DNA repair.

## Discussion

In this study, we used premature spindle extension in HU to assess the genetic basis for S phase checkpoint defects in *STN1* OP cells. This approach was predicated on our recent observations that mutations that suppress *rad53* checkpoint defects in DNA replication control (*mcm2-1*, *mcm5-1*, *dbf4-zn*, *exo1-∆*) co-suppress defective spindle extension, suggesting a mechanistic coupling between these phenotypes (Julius et al. 2019). Based on this previous study, we proposed spindle extension is an indirect consequence of replication fork catastrophes in the vicinity of centromeres. These catastrophes occur due to simultaneously deregulating two key Rad53 effector responses: loss of the check on *ORI* firing, which exacerbates nucleotide depletion, and loss of replication fork stability, which generates ssDNA. A key finding of the work presented here is that spindle extension induced by *STN1* OP in HU is suppressed by the same set of DNA replication and nuclease mutations that suppress *rad53*, indicating gain of *STN1* and loss of *RAD53* deregulate similar processes in DNA replication control. As discussed below, our data cumulatively suggest DNA replication functions for Stn1 likely converge on the MCM complex.

### Relationships between STN1, RAD53 and the DDK in the S phase checkpoint

In *STN1* OP cells, Rad53 exhibits the electrophoretic mobility shift characteristic of Rad53 auto-phosphorylation, indicating checkpoint signaling upstream of Rad53 is not disrupted by excess Stn1 (Gasparyan et al. 2009). Since we show Stn1 likely binds Dbf4, it remains possible that *STN1* OP interferes with the ability of Rad53 to complex with and phosphorylate this effector substrate. Alternatively, *STN1* OP could interfere with the checkpoint indirectly, by circumventing Rad53 regulatory mechanisms. Our observations lead us to favor the latter interpretation, with Stn1 functioning as an accessory replication factor rather than a Rad53 anti-checkpoint. First, *STN1* OP is lethal to *dun1* and *rnr2* mutant strains, suggesting an increased demand on dNTP pools even in the absence of HU. Second, a non-essential role for Stn1 in DNA replication is supported by the observations that *stn1* mutants accumulate ssDNA outside of telomeres and impose a requirement for post-replication DNA repair. Third, *STN1* OP acts as a dosage suppressor of *mcm7-1*. The basis for this suppression remains to be determined. Since *mcm7-1* abolishes transcriptional repression of other *MCM* genes, it is possible overproduced Stn1 may suppress *mcm7-1* through processes other than restoring Mcm7 activity within the Mcm2-7 hexamer. Fourth, the epistasis of the *stn1*^*1−186*^ phenotype in *rad53-21* in *stn1*^*1−186*^* rad53-21* double mutants indicates that Stn1 is necessary to manifest *rad53* HU phenotypes. One genetic interpretation is that—like Dbf4, Sld3, and Exo1—Stn1 is another effector that is negatively regulated by Rad53, with the absence of inhibition leading to deregulated *ORI* firing and fork destabilization at centromeric regions (Supplemental Fig. 2). Whether Stn1 is a direct target of Rad53 in the S phase checkpoint will be important to assess in future studies.

One possibility we addressed in this study is that *STN1* OP might activate *ORI* firing in HU by promoting DDK activity towards MCM substrates. This is because, first, we detected a likely physical interaction between Dbf4 and Stn1, and, second, to our knowledge, only *STN1* OP and *DBF4* OP have been shown to be sufficient to over-ride Rad53 inhibition of *ORI* firing (Gasparyan et al. 2009; Mantiero et al. 2011; Tanaka et al. 2011; Julius et al. 2019). How increased DDK activity circumvents the parallel Rad53 check on Sld3 is not clear. The minimal essential role for the DDK in activating MCM is to relieve an auto-inhibitory activity with the N-terminus of Mcm4 (Sheu and Stillman 2010). However, deletion of this domain (Mcm4^∆74–174^) is not sufficient to bypass the Rad53 check on late *ORI* firing; it is also necessary to simultaneously bypass Rad53 inhibition of Sld3 (Sheu et al. 2016). The same pattern is observed with *mcm5-bob1*, which is also proficient for the Rad53 check on *ORI* firing (Zegerman and Diffley 2010). *DBF4* OP enriches Sld3 and other initiation factors at late firing *ORI*s (Tanaka et al. 2011). Such enrichment could conceivably circumvent the Rad53 block on Dbf4 and Sld3 at the S phase checkpoint.

Although Stn1 may function in a positive-acting manner with the DDK, the findings presented here indicate that the ability of *STN1* OP to force checked *ORI* firing in HU cannot be directed exclusively through the DDK. *dbf4-∆ mcm5-bob1*/*STN1* OP cells still exhibit spindle extension in HU which, from a genetic standpoint, argues *STN1* OP must have an additional target(s). We therefore propose Stn1 acts in a parallel, potentially reinforcing, pathway with the DDK to activate *ORI* firing (Supplemental Fig. 2). This is supported by the observation that *mcm2-1* and *mcm5-1* are the strongest suppressors of HU spindle extension in both *rad53* mutants and *STN1* OP cells, suggesting *STN1* and *RAD53* ultimately converge on MCM. In sum, our genetic analysis of gain and loss of *STN1* function is consistent with Stn1 participating in a nexus of interactions involving MCM/CMG, *POL12*/Polα, and the DDK. During HU challenge, the effect of excess Stn1 within this network is to counteract key aspects of Rad53 DNA replication control. Conversely, when Stn1 fails to act within this network, cell accumulate ssDNA indicative of replicative DNA damage, even in the absence of exogenous replication stress.

### Speculative roles for Stn1 in MCM function

While our study does not address the molecular basis for how Stn1 activates MCM, several observations warrant discussion. In a potentially related manner to budding yeast, OP of Stn1 in human cells stimulates firing of dormant *ORI*s during HU recovery (Wang et al. 2014), while Stn1 depletion decreases *ORI* activation after replication stress (Wang et al. 2012). Additionally, in a recent study human Stn1 was shown to bind to Mcm4 and Mcm7, as well as to Ctf4/And1 (Wang et al. 2019). Ctf4/And1 functions as an adaptor that links Polα to the replisome and also potentially tethers bidirectional replisomes together (Yuan et al. 2019). In yeast, the Ctf4–Polα linkage is preferentially involved, although not essential, for initiating lagging strand synthesis (Porcella et al. 2020). Knockdown of human Stn1 was found to reduce And1 chromatin association during recovery from replication stress, leading to a model where CST provided a backup mechanism to recruit And1/Polα, thereby stimulating initiation of lagging strand synthesis under challenging firing conditions (Wang et al. 2019). Stn1 has also been shown to stimulate Polα priming/catalysis and replication of ssDNA templates in vitro (Goulian and Heard 1990; Nakaoka et al. 2012), and, from our previous work, disruption of Stn1 binding to the Pol12 subunit of Polα rescued *STN1* OP S phase checkpoint defects (Gasparyan et al. 2009). It, therefore, seems likely that there is a conserved role for Stn1 in *ORI* firing that is closely coupled to Pol12/Polα.

If Stn1 plays a conserved role in stimulating Polα activity during stress-related *ORI* firing, how might a connection with MCM be involved? One possibility is that *STN1* facilitates a coupling between CMG activation and Polα recruitment and priming. As revealed in human cells, this may involve bridging interactions between Stn1, MCM and And1/Ctf4 (Wang et al. 2019). Given our finding Stn1 likely also interacts with Dbf4, a related possibility is that Stn1 helps maintain an activated status for MCM. DDK-mediated phosphorylation of MCM is counteracted by the Glc7/PP1 phosphatase, which is recruited to *ORI*s through the Rap1-interacting factor Rif1 (Boos and Ferreira 2019). Thus, Stn1 may be recruited to the replisome not just to facilitate lagging strand synthesis under challenging conditions but also to counteract Rif1 and maintain MCM phospho-activation (Supplemental Fig. 2). Recent evidence suggests DDK activity towards MCM is involved not just in the initial activation of MCM during *ORI* firing, but also in maintaining CMG activity at challenged replication forks (Cabello-Lobato et al. 2021; Dolson et al. 2021). The role of Rad53 in stabilizing replication forks in HU is also be closely coupled to CMG, with Rad53 blocking CMG advance beyond the site of leading strand synthesis during replication stress (Gan et al. 2017; Devbhandari and Remus 2020). Although the Rad53 mechanism enforcing this coupling is not yet clear, Rad53 docking sites within CMG are likely to be involved (Can et al. 2019). We therefore speculate the similarities between gain of *STN1* and loss of *RAD53* encompasses both functional populations of MCM. In this view, Stn1 plays an accessory role in activating MCM conversion to CMG during *ORI* firing and stimulates CMG advance through difficult templates. Disruptions to these functions could lead to the accumulation of ssDNA gaps during S phase and the requirement for post-replication DNA repair we detected in *stn1* mutants.

## Supplementary Information

Below is the link to the electronic supplementary material.

Supplementary file1 Supplemental Figure 1: Two-hybrid interaction between Stn1 and Dbf4. (A) Stn1, but not Cdc13 or Ten1, interacts with Dbf4. Plasmids encoding the indicated genes fused to either the DNA binding domain (DBD, bait) or activation domain (AD, prey) of Gal4 were transformed into strain PJ69-4A containing HIS3, ADE2 and lacZ reporter genes under transcriptional control from the ADH promoter (James et al. 1996). 10-fold serial dilutions of bait-prey transformants were stamped onto Trp-Leu-plates that maintain selection for the two-hybrid plasmids or onto plates that assess activation of individual reporter genes (Trp-Leu-His-25 mM 3AT, Trp-Leu-Ade- and Trp-Leu-/Xgal overlay). Plates were incubated for 3–4 days. Plasmids: Vector (Vec; pACT2.2), pDBD-DBF4 (pCN515), pAD-STN1 (pCN366), pAD-CDC13 (pVL855), pAD-TEN1 (pCN125). (B) Mapping Stn1 regions necessary for two-hybrid association with Dbf4. A series of Stn1 truncations fused to the GAL4 activation domain were assessed for interaction with pDBD-DBF4. Plasmids: pDBD-DBF4 (pCN515), pAD-STN1 (pCN366), pAD-stn1-∆WH1, pAD-stn11-186, pAD-stn11-281, pAD-stn11-371, pAD-stn1173-494, pAD-stn1186-494, pAD-stn1288-494. In this nomenclature, amino acid breakpoints represent residues that are included in Stn1 two-hybrid fragments. (C) Compilation of two-hybrid results. The OB fold domain of Stn1, as well as addition residues on the C-terminal side of the OB fold, appear to constitute the region involved in Stn1 interaction with Dbf4. (TIFF 6247 KB)

Supplementary file2 Supplemental Figure 2. Possible roles for Stn1 as an accessory replication factor. Dashed lines indicate hypothetical roles for STN1 based on published literature and results of this work. See text for relevant citations. While aspects of the modeling are speculative, the rationale is to provide a framework for interpretating how STN1 OP in HU so closely parallels loss of RAD53. 1. Mec1 is recruited to challenged forks in HU and activates Rad53 through Mrc1. Rad53 stabilizes forks through a response in which Rad53 complexes with CMG. This prevents advance of the helicase past the site of leading strand synthesis and prevents exposure of ssDNA on the leading strand template. 2. Given the similarity between STN1 OP and rad53 in HU, one possibility is that Stn1 acts at forks to promote advance through challenging templates. Stn1 could work in concert with Polα/primase to ensure polymerase coupling or to facilitate priming events that allow DNA synthesis to keep pace with CMG. As discussed in the text, Stn1 could also facilitate CMG advance by counteracting PP1 to maintain the phospho-activation of MCM or other kinase substrates. This could be important, for example, for fork restoration mechanisms. 3. Rad53 interrupts the ORI firing program by delaying later-firing (checked) ORIs. This is mediated by Rad53 complexing with and phosphorylating Dbf4, as well as parallel targeting of Sld3. This prevents the phospho-activation of Mcm2–7 and early steps in CMG assembly. 4. STN1 OP over-rides Rad53 inhibition of checked ORIs in HU. As described in the text, our analysis suggests Stn1 activates ORI firing through a nexus of interactions involving the DDK, Pol12, and Mcm2–7. These interactions presumably allow MCM to attain an active configuration at ORIs that is compatible with DNA unwinding, CMG assembly, and initiation of DNA synthesis. (TIFF 810 KB)
