# Supplementary figures and images for: Yeast Stn1 promotes MCM to circumvent Rad53 control of the S phase checkpoint
Source: Curr Genet. 2022 Feb 12;68(2):165–79. doi: 10.1007/s00294-022-01228-0 (PMC8976814; doi:10.1007/s00294-022-01228-0)

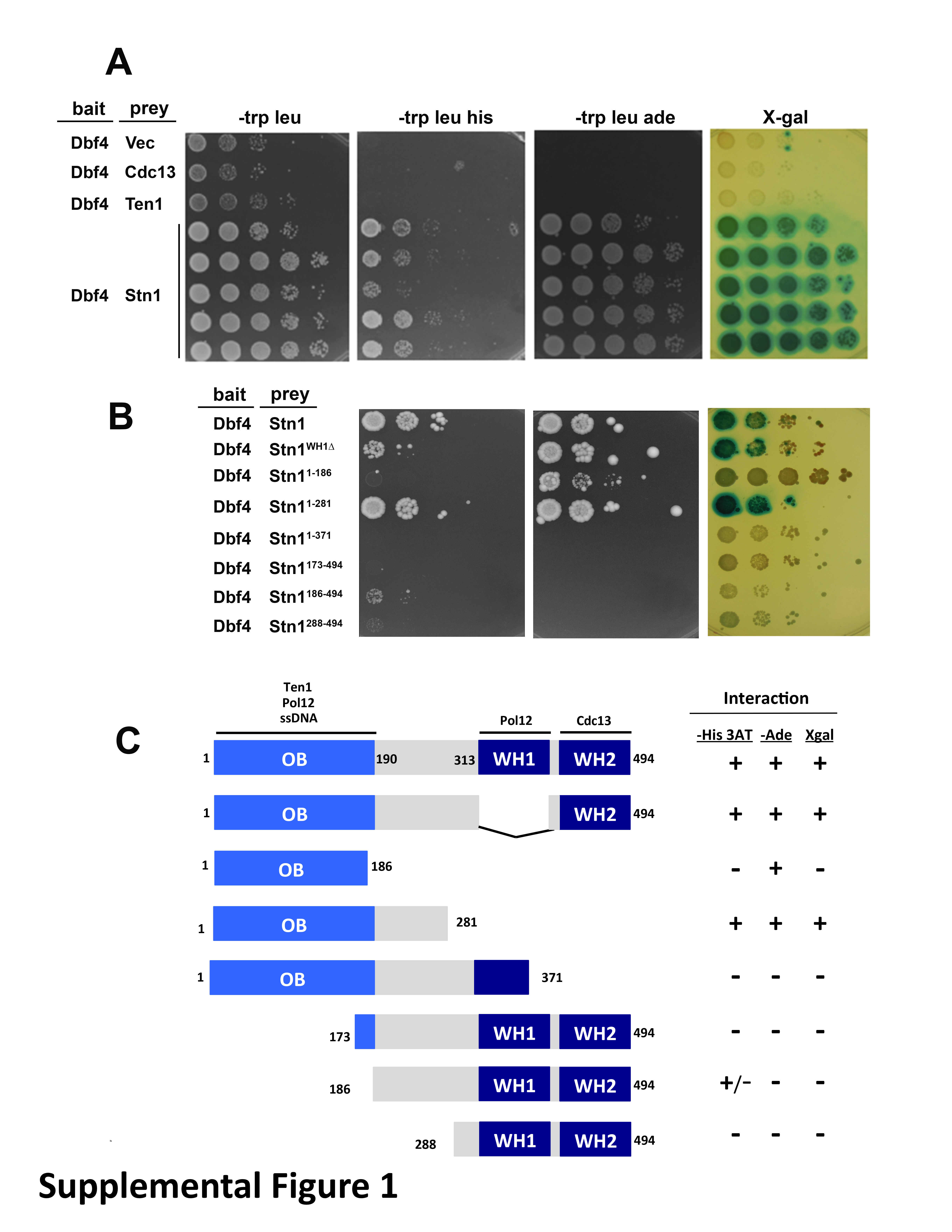

Supplement: Supplementary file 1 — Supplementary file1 Supplemental Figure 1: Two-hybrid interaction between Stn1 and Dbf4. (A) Stn1, but not Cdc13 or Ten1, interacts with Dbf4. Plasmids encoding the indicated genes fused to either the DNA binding domain (DBD, bait) or activation domain (AD, prey) of Gal4 were transformed into strain PJ69-4A containing HIS3, ADE2 and lacZ reporter genes under transcriptional control from the ADH promoter (James et al. 1996). 10-fold serial dilutions of bait-prey transformants were stamped onto Trp-Leu-plates that maintain selection for the two-hybrid plasmids or onto plates that assess activation of individual reporter genes (Trp-Leu-His-25 mM 3AT, Trp-Leu-Ade- and Trp-Leu-/Xgal overlay). Plates were incubated for 3–4 days. Plasmids: Vector (Vec; pACT2.2), pDBD-DBF4 (pCN515), pAD-STN1 (pCN366), pAD-CDC13 (pVL855), pAD-TEN1 (pCN125). (B) Mapping Stn1 regions necessary for two-hybrid association with Dbf4. A series of Stn1 truncations fused to the GAL4 activation domain were assessed for interaction with pDBD-DBF4. Plasmids: pDBD-DBF4 (pCN515), pAD-STN1 (pCN366), pAD-stn1-∆WH1, pAD-stn11-186, pAD-stn11-281, pAD-stn11-371, pAD-stn1173-494, pAD-stn1186-494, pAD-stn1288-494. In this nomenclature, amino acid breakpoints represent residues that are included in Stn1 two-hybrid fragments. (C) Compilation of two-hybrid results. The OB fold domain of Stn1, as well as addition residues on the C-terminal side of the OB fold, appear to constitute the region involved in Stn1 interaction with Dbf4. (TIFF 6247 KB) [file 294_2022_1228_MOESM1_ESM.tiff]

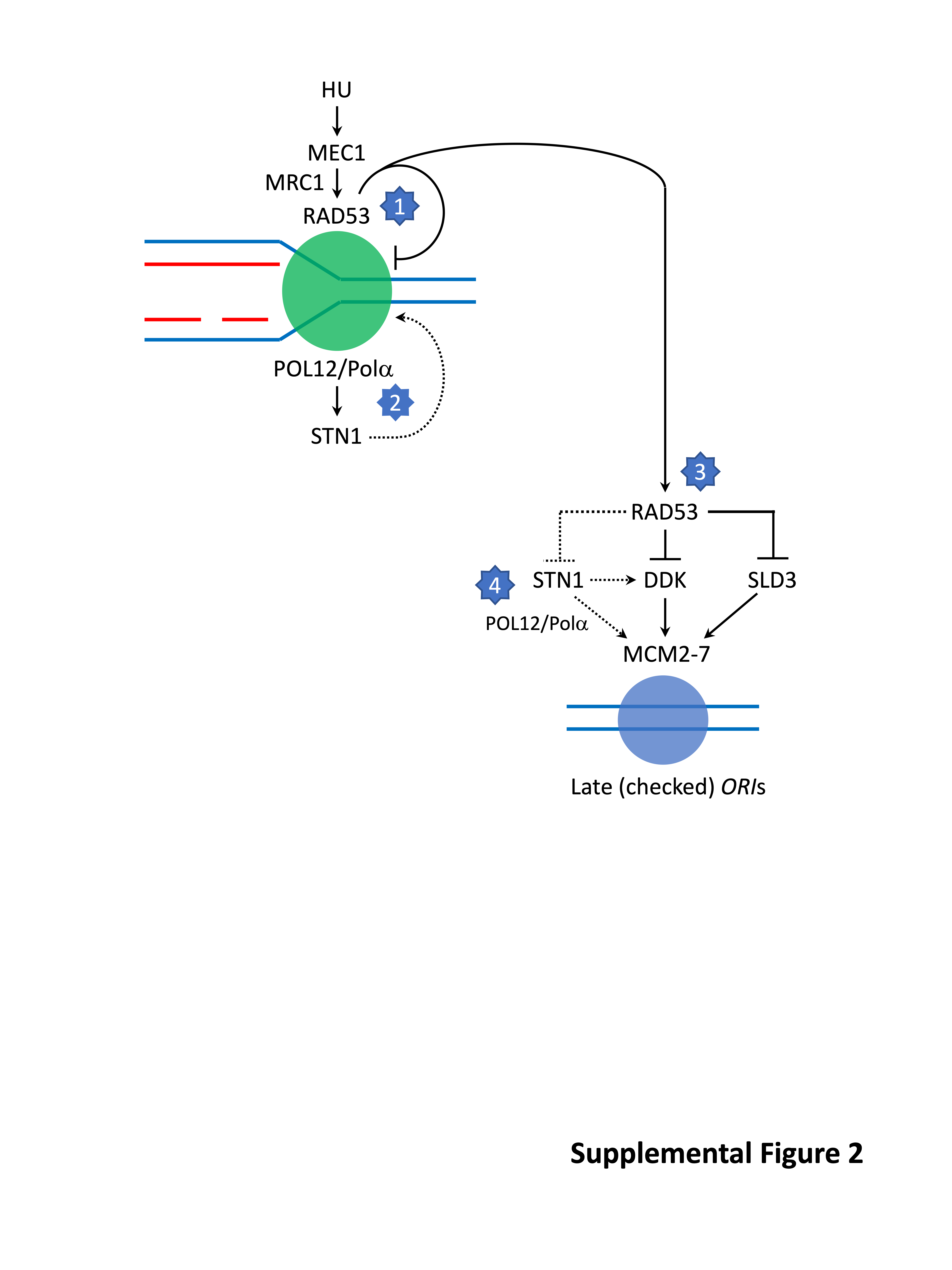

Supplement: Supplementary file 2 — Supplementary file2 Supplemental Figure 2. Possible roles for Stn1 as an accessory replication factor. Dashed lines indicate hypothetical roles for STN1 based on published literature and results of this work. See text for relevant citations. While aspects of the modeling are speculative, the rationale is to provide a framework for interpretating how STN1 OP in HU so closely parallels loss of RAD53. 1. Mec1 is recruited to challenged forks in HU and activates Rad53 through Mrc1. Rad53 stabilizes forks through a response in which Rad53 complexes with CMG. This prevents advance of the helicase past the site of leading strand synthesis and prevents exposure of ssDNA on the leading strand template. 2. Given the similarity between STN1 OP and rad53 in HU, one possibility is that Stn1 acts at forks to promote advance through challenging templates. Stn1 could work in concert with Polα/primase to ensure polymerase coupling or to facilitate priming events that allow DNA synthesis to keep pace with CMG. As discussed in the text, Stn1 could also facilitate CMG advance by counteracting PP1 to maintain the phospho-activation of MCM or other kinase substrates. This could be important, for example, for fork restoration mechanisms. 3. Rad53 interrupts the ORI firing program by delaying later-firing (checked) ORIs. This is mediated by Rad53 complexing with and phosphorylating Dbf4, as well as parallel targeting of Sld3. This prevents the phospho-activation of Mcm2–7 and early steps in CMG assembly. 4. STN1 OP over-rides Rad53 inhibition of checked ORIs in HU. As described in the text, our analysis suggests Stn1 activates ORI firing through a nexus of interactions involving the DDK, Pol12, and Mcm2–7. These interactions presumably allow MCM to attain an active configuration at ORIs that is compatible with DNA unwinding, CMG assembly, and initiation of DNA synthesis. (TIFF 810 KB) [file 294_2022_1228_MOESM2_ESM.tiff]
